# Improving the specimen referral system in Ghana: findings from a landscape assessment

**DOI:** 10.3389/fpubh.2025.1645873

**Published:** 2025-09-02

**Authors:** Christopher Nkrumah, Paa Kobina Forson, Bernard Nkrumah, Richard Owusu, Mohammed Aminu Andrew Musah, Doreenda Enyonam Ahiataku, Gifty Boateng, Pearl Nanka-Bruce, Franklin Asiedu-Bekoe, Horlali Yao Gudjinu, William Addo Mills-Pappoe, John T. Ayivase, Ignatius Nchor Awinibuno, Danielle T. Barradas

**Affiliations:** ^1^Jhpiego Corporation, Accra, Ghana; ^2^African Field Epidemiology Network, Accra, Ghana; ^3^National Public Health and Reference Laboratory, Ghana Health Service, Accra, Ghana; ^4^Public Health Division, Ghana Health Service, Accra, Ghana; ^5^Clinical Laboratory Unit, Ghana Health Service, Accra, Ghana; ^6^Technical Coordination Directorate, Allied Health Department, Ministry of Health, Accra, Ghana; ^7^Division of Global Health Protection, Global Health Center, US Centers for Disease Control and Prevention, Accra, Ghana

**Keywords:** specimen referral, laboratory network, disease surveillance, priority diseases, Ghana, biosafety

## Abstract

**Introduction:**

Ghana's specimen referral system (SRS) is driven by vertical surveillance programs and outbreak response events; the lack of integration limits public health disease surveillance capacity. We assessed the current state of the SRS, the existence of biosafety, biosecurity guidelines, and the turnaround time (TAT) from sample collection to result return.

**Methods:**

We conducted a cross-sectional survey using the African Society for Laboratory Medicine (ASLM) specimen referral tool in nine regions. A total of 265 health facilities were selected using multistage sampling. Surveillance officers, health directors, laboratory scientists, and specimen transporters were purposively selected for interviews. Also, records on SRS performance were reviewed.

**Results:**

A *hub-and-spoke* system was used to transport specimens from collection points to laboratories for the HIV and TB programs. A *two-way system* was used to transport specimens for infectious diseases under surveillance. Within these systems, motorbikes, trucks, and mini-vans were used to transport specimens. Results were tracked using phone calls, referral logs, and the Surveillance Outbreak Response Management and Analysis System (SORMAS); results were mainly returned electronically (61.8%; 123/199). Health management teams (HMT) at regional and district health directorates (DHDs) had packaging guidelines or standard operating procedures (SOPs) for biological specimens (66%; 88/133) and had trained healthcare workers on how to transport specimens (59%; 79/133). Only 28% (55/199) of referring facilities had these guidelines/SOPs, and 45% (90/199) had at least one health worker trained in specimen packaging. Futhermore, the availability of triple-packaging materials was limited at all levels of the healthcare system, transport companies did not have guidelines/SOPs for handling specimens, and transporters were not trained on specimen handling. All reference laboratories had the necessary guidelines/SOPs. The average TAT for all specimens was 12 days, with delays occurring at collection facilities.

**Discussion:**

Ghana has many pathways for transporting specimens within the disease surveillance system at no cost to patients; however, notable weaknesses exist. Inadequate resources for transportation and lack of adherence to biosafety guidelines remain major challenges. These inefficiencies in the SRS could impact the timely detection and response to health threats and may increase the risk of diseases spreading within and beyond Ghana's borders.

## 1 Introduction

The International Health Regulations (IHR) 2005, in consonance with the Global Health Security Agenda, mandates that signatory member states develop strategies to strengthen public health responses to priority diseases. Specifically, countries are required to develop, strengthen, and maintain the capacity to prevent, detect, assess, notify, and respond to public health emergencies (1). The occurrence of several recent pandemics has shown the impact these infections pose to both human health and world economies (2). In response, many countries within the African subregion are making concerted efforts to enhance their public health systems, including the strengthening of laboratory networks (3). A well-functioning laboratory network is critical for the timely detection of priority diseases and depends on a robust specimen referral system (SRS), ensuring biological specimens are safely transported from peripheral facilities to designated testing laboratories (4).

In Ghana, many policies and strategies have been implemented to achieve compliance with IHR (2005) requirements, including the adoption of Integrated Disease Surveillance and Response (IDSR) (5), enactment of the Public Health Act, 2012 (Act 851) (6) and participation in the World Health Organization (WHO) Joint External Evaluation (7, 8). The Ghana Health Service (GHS) further collaborates with several local and international partners to support the planning and implementation of a more efficient SRS within the laboratory network to rapidly confirm epidemic-prone diseases. For instance, the United States Centers for Disease Control and Prevention (US CDC) supports the transportation of influenza-like illness specimens from sentinel sites to the National Influenza Center (NIC) located at the Noguchi Memorial Institute for Medical Research (NMIMR), Accra, Ghana. Additionally, WHO funds the referral of polio and measles specimens to the National Public Health and Reference Laboratory (NPHRL), Korle Bu, Accra, Ghana.

In addition to managing these SRS programs, the government oversees the sample referral of other priority pathogens such as anthrax and cholera. Despite these efforts and immense contributions from the partnerships, the significant delays in the confirmation of recent outbreaks of Lassa fever, Mpox, yellow fever, and COVID-19 highlight the need to make further efforts to strengthen the health system to better mitigate infectious disease threats. Many studies assessing the state of Ghana's health emergency preparedness and response capacity have identified the lack of coordination, inadequate logistics, and insufficient funding of the SRS and have emphasized the importance of an efficient SRS in enhancing access to quality testing and improving laboratory surveillance (9–13). Moreover, international guidelines on specimen transport recommend regular review of the SRS to identify priority areas for improvements; however, such evaluations are limited in Ghana (8). This study, therefore, aimed to understand the existing SRS and assess its functionality with respect to turnaround time (TAT) and compliance with biosafety and biosecurity measures. The findings will guide the development of more efficient SRS to enhance the rapid detection of pathogens and support timely public health interventions, including containment, mitigation, and prevention strategies.

## 2 Methods

### 2.1 Study design and setting

A cross-sectional survey was conducted in 265 selected health facilities and eight transport companies in 45 districts across nine regions in Ghana from November to December 2023 by Jhpiego and Ghana Health Service African Field Epidemiologists Network (AFENET).

### 2.2 Sampling

**Figure 1:**
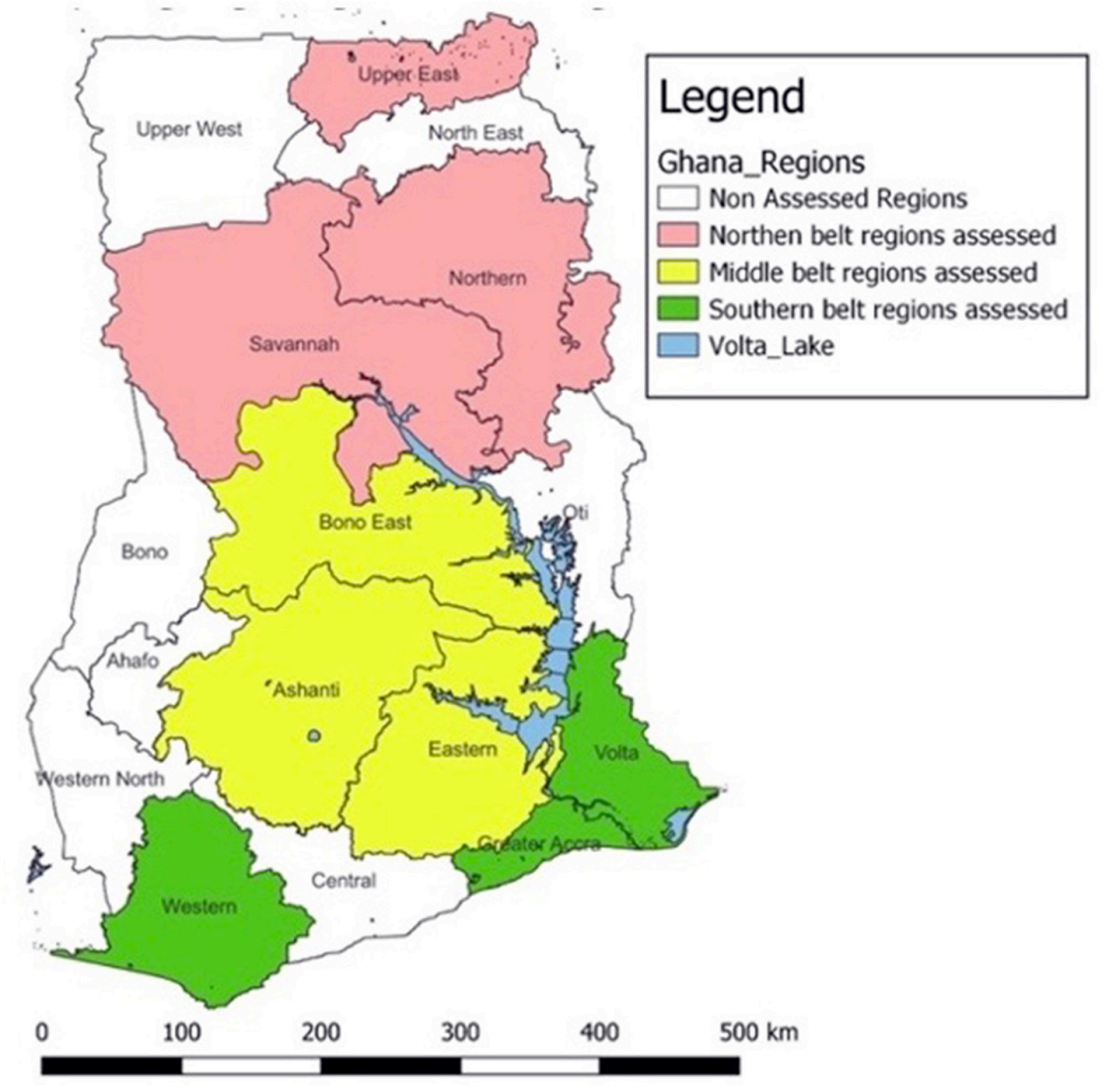
Map of assessed regions across Ghana.

A multistage sampling technique was used to select regions, districts, and health facilities for the assessment. For the purpose of this study, the country was divided into three geographical zones: Northern, Middle, and Southern (Figure 1). Geographic stratification was employed as a design approach to ensure national-level representation of Ghana's SRS. Simple random sampling using the lottery method was used to select three regions from each zone, adding up to 9 regions. In selected regions, all Regional Health Directorates (RHDs) and teaching hospitals were included in the assessment. RHDs are primarily responsible for the coordination and management of specimen referral at the district and sub-district levels, while teaching hospitals serve as main referral hospitals in the regions.

In the selected regions, districts with high Outpatient Department (OPD) attendance facilities were selected. In all, 45 districts were included in the assessment. All District Health Directorates (DHDs) of selected districts were included in the assessment. This is because DHDs are responsible for the coordination and management of SRS at the district level. Also, OPD attendance was used because it remains a frequently essential indicator in disease surveillance and control programs. Focusing on high-OPD facilities allows the assessment to acquire a more comprehensive picture of the SRS performance.

In addition, hospitals, health centers, and Community-based Health Planning and Services (CHPS) with high OPD attendance were also selected (for hospitals: monthly OPD attendance ≥500; health centers ≥200, and CHPS ≥150). All transport companies transporting specimens in the various sub-districts, districts, and regions were also included in the assessment. Lastly, all reference laboratories in the regions were also included.

Surveillance and disease control officers, health directors, and laboratory scientists at the district, regional, and national levels were purposively selected for interview based on their respective roles in the SRS at each level.

### 2.3 Data collection

Data was collected over 4 weeks during November–December 2023. A national Technical Working Group (TWG) was formed to review and adapt the African Society for Laboratory Medicine (ASLM) specimen referral tool to fit Ghana's context (14). This tool is semi structured (allowing the capture of both quantitative and qualitive data from participants) and comprised five questionnaires targeting the following areas: (1) *MoH or Health Management Team questionnaire* captured information on the landscape, laboratory network/infrastructure, referral network structure, policies, and logistics; (2) *reference laboratory or Hub questionnaire* captured information on testing capacity, laboratory network structure, quality of specimen, standard operating procedures (SOPs), and biosafety and biosecurity; (3) *referring facility questionnaire* captured information on systems/communication, type of samples referred, and tracking system; (4) *disease program questionnaire* captured summary of diagnostic capacity for specific programs such as TB and HIV and; (5) *transporter questionnaire* captured information on specimen transportation and results reported directly from the transporter or the manager.

Eighteen data collectors were recruited for the survey and assigned to teams based on geographical zones. A 3-day residential training was organized for the data collectors to deepen their understanding of the methods and procedures for conducting the survey. The training focused on the effective use of the KoboCollect application (https://kf.kobotoolbox.org), covering its configuration and navigation to ensure reliable digital data capture. Participants were also guided through the content and structure of the data collection tool. Core ethical principles, including data integrity and confidentiality, were emphasized and aligned with global best practices. Furthermore, sessions on effective interviewing techniques were conducted to foster meaningful engagement with respondents. The cadre of staff selected for this training were medical laboratory scientists and disease control officers with in-depth knowledge and experience in specimen management. These participants were drawn from the GHS, Christian Health Association of Ghana, research institutions, and the Veterinary Services Directorate. Each team comprised two data collectors and a team supervisor to conduct face-to-face interviews in their assigned zones using KoboCollect during November–December 2023. Additionally, monthly records (January 2023–November 2023) on SRS performance, such as TAT, were reviewed. In total, 273 respondents were interviewed during the survey. These interviewees comprised a broad array of key management members at the regional, district, and sub-district levels of the healthcare system. Technical officers at the various healthcare facilities were also interviewed. These included medical laboratory scientists, surveillance officers, and program coordinators. In addition, the leadership of teaching hospitals, public health reference laboratories, Allied Health Directorate, Veterinary Services Directorate, and clinical laboratory units were also interviewed. Management officials from courier and public transport services responsible for specimen referral were also interviewed.

### 2.4 Data analysis

Electronic data was exported from KoboCollect into Microsoft Excel 2019 and analyzed using Stata 17/MP (StataCorp, USA). Consistency and appropriateness of responses were verified through follow-ups with regional and district health facilities. Descriptive statistics, including frequencies and proportions, were used to summarize the data. Data from health facilities in the nine regions from the three zones were aggregated for analysis, ensuring a comprehensive national-level analysis of SRS performance.

### 2.5 Ethical considerations

The study protocol was reviewed and approved by the GHS Ethics Review Committee (GHS-ERC: 015/12/23), Johns Hopkins BSPH (IRB No: 24573/MOD4630), and US CDC (Accession #: CGH-CSIB-7/16/20-95e37). Written approval was also sought from the Director General of GHS, as well as regional and district health directorates of the assessed regions.

## 3 Results

In all, 265 health facilities were covered. These facilities comprised teaching hospitals (4), DHD/RHDs (54), district and regional hospitals (84), health centers (70), CHPS (45), reference laboratories (5), and the national disease-specific program offices (HIV/TB) (3) (Table 1). Eight transport companies responsible for specimen transportation were also included (Table 1).

**Table 1:** Number of health facilities and institutions involved with SRS assessed.

**Region**	***N***	**Teaching hospital**	**RHD/DHD**	**Hospital**	**Health centers**	**CHPS**	**Reference lab**	**National disease-specific program**	**Transport company**
Ashanti (MZ)	43	1	9	16	9	7	1	0	0
Bono East (MZ)	25	0	5	6	8	4	0	0	2
Savannah (NZ)	11	0	3	2	4	2	0	0	0
Eastern (MZ)	44	0	9	15	11	8	0	0	1
Greater Accra (SZ)	42	1	7	13	8	6	3	3	1
Northern (NZ)	28	1	6	9	7	5	0	0	0
Volta (SZ)	27	1	5	8	8	5	0	0	0
Western (SZ)	29	0	5	9	8	4	1	0	2
Upper East (NZ)	24	0	5	6	7	4	0	0	2
Total	273	4	54	84	70	45	5	3	8

MZ, Middle Zone; NZ, Northern Zone; SZ, Southern Zone.

Of the 265 health facilities, 199 were referring facilities and 133 were Health Management Teams (HMT; Table 2). One representative from each referring facility and management team was interviewed. In referring facilities, data was collected on specimen collection and transport, capacity of health workers to provide SRS services, and compliance with biosafety and biosecurity. Facility records were also reviewed for specimen TAT. At the management level, data was collected on the landscape, laboratory network/infrastructure, referral network structure, logistics, and compliance with biosafety and biosecurity.

**Table 2:** Summative reasons for prolonged TAT.

**TAT timelines**	**Reasons for delay**
Collection to pick-up	Shortages of essential materials like containers, collection kits, and cold chain facilities, and inadequate vehicles
Pick-up to lab	Unreliable transportation systems, poor road networks, limited vehicles, and geographic barriers such as hard-to-reach facilities
Results to delivery	Insufficient training, weak coordination between collection points and laboratories, lack of standardized systems (Laboratory Information Management System), and poor network connectivity

### 3.1 Laboratory network in Ghana

**Figure 2:**
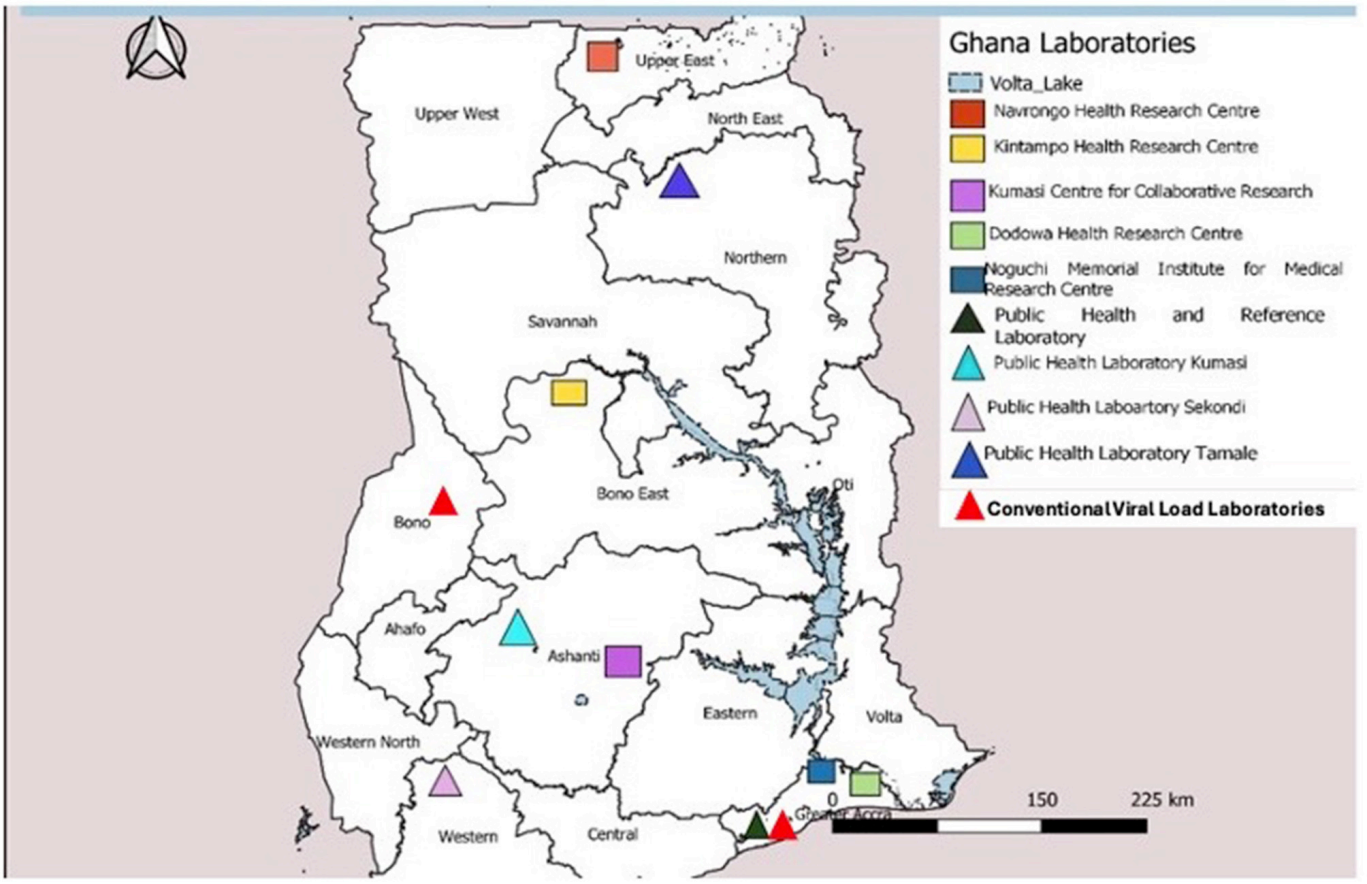
Network of public health, academic, and research laboratories in Ghana, 2023.

Ghana has a well-defined tiered laboratory network consisting of one national and three zonal public health reference laboratories, teaching hospitals, and regional, district, and health center laboratories. The national and zonal public health laboratories fall under the Department of Public Health Laboratories within the Public Health Division of GHS. The GHS is an agency under Ministry of Health responsible for the implementation of national health policies. All other clinical laboratories are coordinated by the Clinical Laboratory Unit within the Institutional Care Division of GHS. Academic research laboratories, such as Noguchi Memorial Institute for Medical Research (NMIMR), affiliated to the University of Ghana and Kumasi Center for Collaborative Research (KCCR) under Kwame Nkrumah University of Science and Technology, support surveillance activities in the country. The national laboratory network has advanced capability to test priority diseases under surveillance and vaccine-preventable diseases at the NPHRL, NMIMR, and KCCR (Figure 2).

### 3.2 Snapshot of the specimen referral system

**Figure 3:**
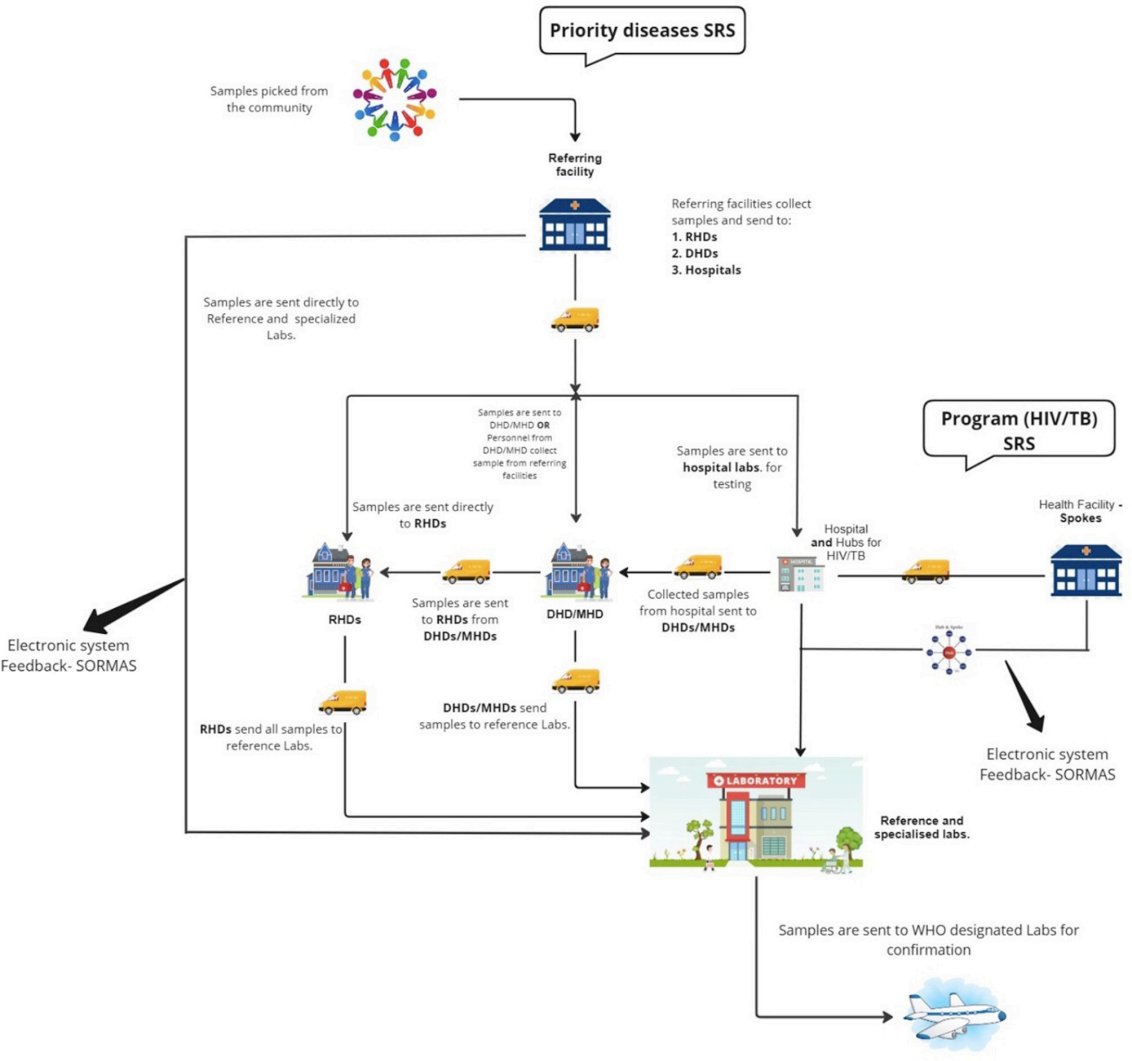
Snapshot of the specimen referral system in Ghana, 2023.

Several pathways were utilized for specimen referral depending on the location of the collection point and the type of specimen involved (Figure 3). For priority diseases or diseases under surveillance, including polio, meningitis, yellow fever, and measles, about 53% (105/199) of health facilities (CHPS compounds, Health Centers, and hospitals) sent specimens to the DHD where they were batched for onward submission to reference or specialized laboratories for testing on *ad hoc* basis. Specimens were labeled with identifiers such as epidemiology number, type of specimen, and address of the laboratory. Motorcycles were typically used to transport specimens from the lower facilities to the next tier(s). The majority of specimens (86%; 171/199) sent to the DHDs were transported by healthcare providers via public vehicles (62%; 82/133). Other facilities (21%; 21/199) sent specimens directly to the reference laboratory for testing or were sent to storage points at the RHD for transport to the testing laboratories (18%; 36/199). Results were tracked via phone calls, referral logs, and the Surveillance Outbreak Response Management and Analysis System (SORMAS). Results were mainly returned electronically (61.8%; 123/199) in addition to using paper-based forms (32.2%; 64/199). The NPHRL also shipped selected biological specimens to WHO-accredited laboratories such as the Pasteur Institute in Dakar for confirmation.

For TB and HIV, all respondents indicated that specimens were transported using a hub and spoke system based on geographic proximity. This model connects facilities with varying levels of diagnostic capacities, where spoke facilities with limited capacity for a particular test refer specimens to “hubs” which are typically well-equipped for testing. TB specimens were collected from the peripheral facilities (spokes) and transported to the “hub” laboratory for testing. Specimens received for tests that could not be performed at the receiving “hub” laboratory were subsequently transferred to the next highest level laboratory that could perform more complex testing, such as testing for extensive drug resistance (XDR) in TB specimens. Results were primarily returned electronically through the TB GxAlert^®^ (SystemOne, USA) in addition to phone calls and paper-based systems. Specimens were tracked using phone call follow-ups and referral logbooks. For HIV viral load (VL) and early infant diagnosis (EID), specimens were sent to strategically created laboratory hubs for testing. There were 137 GeneXpert sites providing HIV VL and or EID services across the country at the time of the assessment. Two sites, Sunyani and Korle Bu Teaching Hospitals, also provided HIV VL and EID testing using conventional polymerase chain reaction.

### 3.3 Turnaround time for SRS in Ghana

**Figure 4:**
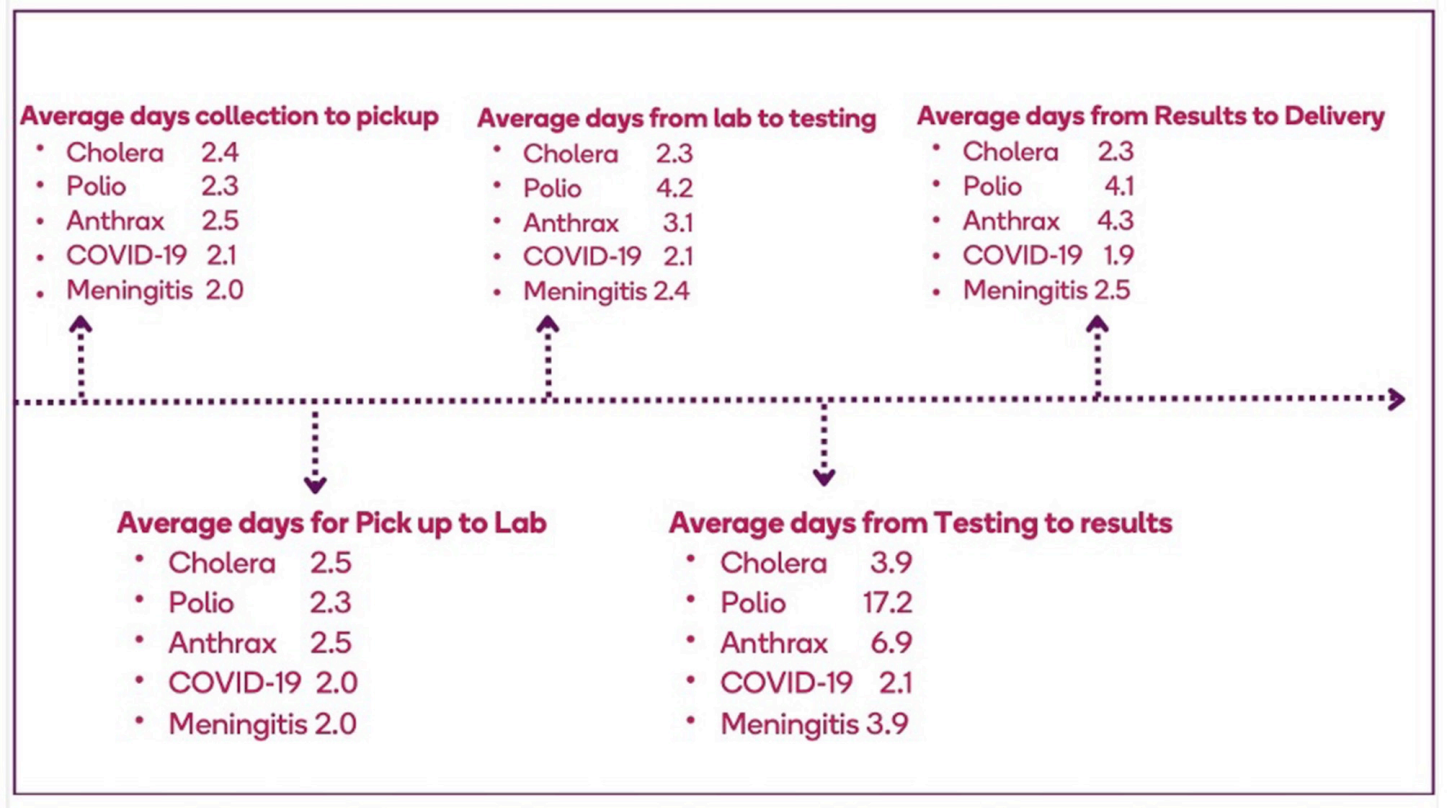
Average turnaround time for specimens that enter the specimen referral system in Ghana, 2023.

Differences were observed with respect to the overall TAT for specimens collected and tested for different pathogens by the system. For example, for testing of a suspected cholera specimen, an average of 12 days was spent from specimen collection to results delivery, with delays occurring at the collection points (up to 4 days). The average TAT for a polio specimen was 20–30 days. An average of 15 days was spent from the collection of an anthrax specimen to the results delivery. COVID-19 and meningitis, had an average of 7 and 12 days between specimen collection to results delivery, respectively (Figure 4). The prolonged TAT for specimen transport was influenced by multiple factors at collection points, including shortages of essential materials like containers, collection kits, and cold chain facilities, which delayed specimen collection and handling (Table 2). Unreliable transportation systems, particularly in rural areas, further contributed to delays, as poor road networks, limited vehicles, and geographic barriers made timely specimen delivery difficult. Additionally, inadequate funding, often dependent on unpredictable sources, hampered transport logistics, affecting vehicle availability and fuel supplies. Unequal and inappropriate distribution of workforce, insufficient training, and weak coordination between collection points and laboratories exacerbated the delays.

### 3.4 Compliance with biosafety and biosecurity

An estimated 66% (88/133) of health management teams at the RHD/DHD had the packaging guidelines or SOPs for biological specimens; 59% (79/133) of RHD/DHD health management teams provided training to RHD/DHD workers on proper handling and transportation (Table 2). However, only 28% (55/199) of referring facilities had and were aware of these packaging guidelines or SOPs, and less than half (45%: 90/199) of health workers in these facilities were trained on the proper collection, handling, and packaging of specimens. Triple-packaging materials were not readily available (43%: 57/133) for specimen transport by health management teams for specimen transportation. None of the transport companies had packaging guidelines or SOPs for biological specimens, and transporters were not trained on the proper collection, handling, packaging, or transportation of specimens. Conversely, all reference laboratories had packaging guidelines/SOPs for biological specimens, with 70% (7/10) of the reference laboratories have had at least one health worker adequately trained on how to properly collect, handle and package specimens and 70% (7/10) reference laboratories consistently have triple-packaging materials (Table 3).

**Table 3:** Compliance with biosafety and biosecurity.

**No**.	**Biosafety and security indicators**	**DHMT/RHD *N* = 133**	**Referring facility *N* = 199**	**Transport companies *N* = 8**	**Reference laboratory *N* = 10**
1	Availability of packaging guidelines or SOPs for biological specimens	88 (66)	55 (28)	0 (0)	10 (100)
2	Workers adequately trained on how to properly collect, handle and package specimens	79 (59)	90 (45)	0 (0)	7 (70)
3	Transporters adequately trained on how to properly handle and transport specimens	53 (40)	90 (45)	0 (0)	4 (40)
4	Triple-packaging/Packaging material consistently and widely available	57 (43)	83 (42)	0 (0)	7 (78)
5	Trained on biosafety or quality-related issues for transport of specimens	79 (59.4)	83 (33)	4 (50)	7 (70)

Referring facility = Hospitals + CHPS + Health Centers; Specimen transporters = Hospitals + CHPS + Health Centers + DHD/RHD (2 facilities do not transport specimen); Health management facilities = DHD + RHD; (Referring facilities were also responsible for the transport of samples).

## 4 Discussion

This study set out to understand the current specimen referral system used by the GHS in Ghana and identify priority areas for optimization. An efficient SRS is a linchpin for maintaining a robust disease surveillance system as it bridges testing deficits within a laboratory network and increases access to laboratory services (15). The recommended approach by the 2017 Global Laboratory Initiative to SRS implementation is one that supports an integrated rather than a fragmented model (14). However, our study revealed that the SRS for priority diseases in Ghana, excluding HIV and TB, is unstructured and uses several different transportation pathways. For instance, specimens for epidemic-prone diseases are transported from peripheral facilities to DHD, RHD, or sometimes directly to designated national laboratories, depending on the region, using a variety of carriers. Though public transport is mostly used for specimen transportation within the various pathways, there was no evidence of any performance contract between the health facilities and designated transport providers. The lack of contractual agreements may contribute to delays in sample transportation, compromised specimen integrity, and improper handling of potentially infectious specimens by the transporters, creating an opportunity for specimen contamination and exposure/infection among those coming in contact with the specimens, including the shipper (16). The lack of contracts in sample transport causes delays due to unclear responsibilities and lack of commitments. Without explicit agreements, there is no clear accountability for timely pickups and deliveries, which often leads to decreased prioritization. The lack of contracts also limits quality control and enforcement measures, increasing the possibility of mishandling specimens or delays in obtaining transportation.

Specimens were also transported on-demand basis from the health facilities; thus, specimens were picked up only when they were collected, without fixed schedules. Though this approach can be well-coordinated to enhance efficiency, especially for surveillance specimens, the increasing need for a high volume of both clinical and surveillance specimens to be referred to higher-level laboratories due to limited testing capacity at the lower levels underscores the critical importance of establishing a dedicated system. Countries such as Uganda and Tanzania, which generate large volumes of specimens for priority disease testing, have a dedicated logistics system for specimen transportation (17). Such a system not only facilitates efficient communication and logistics but also ensures that these specimens are transported as scheduled.

In Ghana, TB and HIV programs utilized a hub and spoke model where peripheral laboratory facilities were linked to a hub based on geographic proximity. Spokes, therefore, sent specimens directly to their assigned hubs for testing. TB and HIV specimens were transported separately from other epidemic-prone diseases due to varying levels of coordination between the disease-specific programs. This parallel system can lead to suboptimal use of scarce resources and duplication of efforts. As previously documented in Mali, weak SRS coordination not only increased operational costs but also hindered the overall efficiency of public health initiatives (18). Many other countries, such as Nigeria, Ethiopia, Uganda, and Guinea, have established structured coordination mechanisms to facilitate the implementation of an integrated specimen referral system (15, 19, 20). The need for collaboration between the government, donors, and partners to build consensus to unify the SRS to manage all specimen types and ensure strong coordination at all levels cannot be overemphasized.

To operate efficiently, SRS should deliver specimens to the testing laboratory in a timely manner while maintaining specimen integrity. TAT is one of the most visible indicators of an effective SRS and is frequently used as a critical performance metric. According to the current Ghana IDSR technical guidelines, specimens related to epidemic-prone diseases such as cholera and meningitis should be transported to laboratories within 24 h of collection, and test results returned within 7 days (5). However, these benchmarks were not universally achieved. TAT can be influenced by factors related to both transportation and diagnostic methods used for the pathogens of interest. In the case of polio, viral isolation typically takes 10–14 days (21). In this study, TAT for priority diseases was unusually prolonged. It took an average of 4 days for specimens to be transported from the facilities to testing laboratories. TAT for meningitis, a disease that constitutes a medical emergency and requires prompt diagnosis and confirmation for clinical management and public health action, was 12 days. This is inconsistent with WHO standards, which recommend that the transportation of cerebrospinal fluid specimens occur within 24 h and the referral laboratory returns results in 5 days (22). This finding is further supported by a study conducted in Yendi, Northern Region, and Ghana where specimens took an average of 3 days to reach the zonal public health reference laboratory in Tamale (23). Plausible explanations for this significant delay were transportation challenges such as unavailability of vehicles, inadequate funds for transportation, and a lack of logistics, such as transport materials, containers, and specimen carriers for referral. The longer wait times for shipping specimens from the health centers that may not have storage facilities can compromise specimen integrity and affect testing at the referral laboratories. In contrast, Burkina Faso has seen significant improvements in the timely referral of specimens for severe acute respiratory illness (SARI). This was accomplished by stockpiling logistics and engaging a dedicated courier agency with clear performance metrics to transport the specimens to the National Influenza Center. About 95% of specimens were delivered within 24 h of pick-up, with no packages lost in the process (24).

Another critical component of a functional SRS is biosafety and biosecurity. Adherence to biosafety and biosecurity not only ensures that specimens are not damaged or contaminated during transport but also that personnel involved in the collection, packaging, and transporting, and the environment are protected from potential danger. The assessment performed in this study showed that adherence to biosafety and biosecurity was generally low, especially at the lower-tier facilities where most of the specimen collection and initial packaging for transport occur. Biosafety was assessed based on WHO criteria for the transportation of infectious pathogens (16). This includes the use of a triple packaging system, leak-proof containers, absorbent material, biological risk labels, and gloves. Most of the facilities improvised triple packaging, but not to the exact requirement of the WHO. This poses a public health threat not only to local populations but to the world, as pathogens can rapidly transcend borders. Factors contributing to the low compliance included inadequate enforcement of the IDSR guidelines, erratic supply of standard materials, equipment, and inadequate training of transport providers.

To optimize the SRS, the following recommendations should be prioritized;

- Develop an integrated national specimen referral system capable of transporting all specimen types with a decentralized level of coordination.
- Adopt a hub and spoke model for all priority diseases, as utilized for TB and HIV, to streamline logistics and reduce transportation cost and TAT.
- Conduct routine biosafety training for health workers and transport personnel, especially at the lower-tier level.
- Enforce WHO-compliant triple packaging standards and provide regular supply of leak-proof containers and protective equipment at all levels.
- Leverage digital tools for specimen tracking, communication, and data management to improve transparency and responsiveness.
- Develop and enforce performance-based contracts with transport providers to ensure accountability, timely delivery and proper specimen handling.

## 5 Conclusion

This assessment demonstrated that Ghana has many pathways for specimen referral with TB and HIV programs utilizing the hub and spoke model. This existing model can be leveraged to establish a decentralized, integrated network covering all specimen types and pathogens. This will allow the SRS to focus on the delivery of specimens to strategically located hubs that are closer to spokes, reducing transport costs and TAT. With this system, specimens requiring confirmatory testing and those with high-priority diseases can be sent from these regional hub laboratories more efficiently and cost-effectively.

## 6 Limitations

While this assessment of the specimen referral system was comprehensive, the variability in referral practices across institutions complicates standardization and comparison of results. It is, therefore essential to establish clear, standardized protocols for specimen collection, processing, and referral across all facilities. This approach will enhance consistency and improve the reliability of future assessments.

## Data Availability

The raw data supporting the conclusions of this article will be made available by the authors, without undue reservation.
